# PdAu Nanosheets for Visible-Light-Driven Suzuki Cross-Coupling Reactions

**DOI:** 10.1021/acsanm.2c03216

**Published:** 2022-10-24

**Authors:** Éadaoin Casey, Justin D. Holmes, Gillian Collins

**Affiliations:** † School of Chemistry, University College Cork, Cork T12 YN60, Ireland; ‡ AMBER Centre, Environmental Research Institute, University College Cork, Cork T23 XE10, Ireland

**Keywords:** two-dimensional nanomaterials, palladium gold alloy nanosheets, visible light, photocatalysis, plasmonic, Suzuki reaction

## Abstract

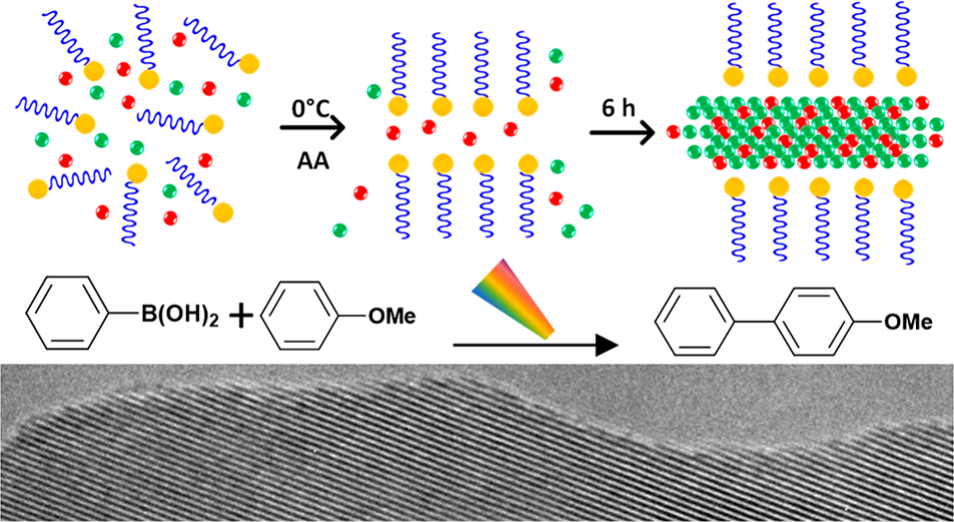

Combining a two-dimensional (2D) morphology and plasmonic photocatalysis represents an efficient design for light-driven organic transformations. We report a one-pot synthesis of surfactant templated PdAu nanosheets (NSs). Transmission electron microscopy (TEM) and X-ray photoelectron spectroscopy (XPS) analyses show the formation of 2D PdAu structures was initiated through nanoparticle seeds dispersed in the alkyl ammonium salt surfactant which acted as a template for the growth into NSs. The PdAu NSs were used for visible-light-enhanced Suzuki cross coupling. The PdAu bimetallic NSs outperformed monometallic Pd NSs and commercial Pd/C in room-temperature Suzuki cross-coupling reactions. The high catalytic activity is attributed to a combination of the 2D morphology giving rise to plasmon-enhanced catalysis and a high density of surface atoms, the electron-rich Pd surface due to alloying, and the presence of weakly bound amines. A comparative study of surfactant-assisted NSs and CO-assisted NSs was also carried out to assess the influence of surface ligands on the catalytic and photocatalytic enhancement of NSs with similar morphology. The surfactant-assisted NSs showed substantially superior performance compared to the CO-assisted for room-temperature Suzuki coupling reactions.

## 1 Introduction

Noble metals are ubiquitous in catalytic applications from chemical synthesis to energy storage and conversion.^1−3^ The use of two-dimensional (2D) metal nanosheets (NSs) for catalysis has attracted attention due to their unique chemical, physical and surface related properties.^1,4,5^ As catalytically active sites are located on the surfaces or edges of catalysts, the presence of a high proportion of exposed atoms and large interfacial area of 2D metal NSs enables them to operate with high atom utilization efficiency, particularly advantageous to noble metal catalysts.^6,7^ 2D metal NSs have been used for water splitting, CO_2_ reduction, and organic synthesis reactions.^8,9^ Pd NSs prepared using layered double hydroxide templates display nearly 40% higher turnover frequency compared to Pd nanoparticles (NPs) for the hydrogenation of nitrobenzene to aniline.^10^ Rh NSs exhibited remarkably higher catalytic activity in the hydrogenation of phenol and cyclohexane compared to commercial Rh/C and PVP-Rh NPs and improved selectivity for the aldehyde product in the hydroformylation of 1-octene.^11^

The intrinsic catalytic activity of noble metals such as Pd can also be significantly increased through alloying with plasmonic metals such as Au and Ag, by exploiting the local surface plasmon resonance (LSPR) of these nanostructures.^12−14^ The development of noble metal catalysts with visible light enhancement has seen much interest in recent years with Pd and Pd alloy nanostructures used for an extensive range of organic transformations driven by visible light such as coupling reactions, hydrogenation and oxidation.^15−17^ The main advantages of using light-harvesting catalysts are lower reaction temperatures compared to conventional thermal reactions, enhanced reaction rates, and improved selectivity,^18^ enabling more energy efficient and greener routes to chemical synthesis. The design of light harvesting heterogeneous catalysts is typically based on plasmonic metal–semiconductor junctions or multimetallic nanostructures consisting of a plasmonic component (usually Au and Ag) and a catalytic component working synergistically, such as alloys or core–shell nanostructures.^19,20^ The ability to combine plasmonic enhancement with a 2D catalyst morphology represents an effective design for energy efficient catalyst driven by visible light. The LSPR peak of Pd is typically located in the ultraviolet spectral range, but 2D Pd NSs have a LSPR that resides in the visible region of the spectrum.^21^

The synthesis of 2D metallic NSs is challenging due to the tendency of metals to form three-dimensional close-packed structures, but there have been advancements in this field in recent years.^22^ Zheng et al.^21^ were the first to report ultrathin Pd NSs with a thickness of 1.8 nm using CO as a structure directing agent. CO is highly effective in controlling the anisotropic growth of 2D metal NSs due to the strong adsorption of CO molecules on the Pd(111) planes which restricts growth along the [111] direction. CO can be replaced with W(CO)_6_ which conveniently removes safety and toxicity issues associated with gaseous CO.^23^ The CO-assisted synthesis method has also been applied to the formation of Pd alloy NSs such as PdFe, PdCo, PdNi, PtCu and PtPdAg.^24−26^ While a CO-assisted route to 2D metal NSs is undoubtedly convenient for high yield and relatively low cost synthesis, a major drawback of using CO as a structure directing agent is that the surfaces of the NS are highly passivated which can be unfavorable or even detrimental for catalytic applications. Xu et al.^27^ demonstrated the bottom-up synthesis of Pd NSs with controlled surface facets through a template-assisted solution-phase growth. By understanding the role of capping ligands, they can be tailored to enhance reactivity of metal nanostructure catalysts, which has also been a topic gaining attention. Yang et al.^24^ showed that replacing oleic acid ligands on CuPd NSs with ethylenediamine ligands led to a highly active catalyst for formic acid oxidation. Amine groups resulted in electron donation to the CuPd interface and is beneficial for the absorption of electron-deficient reactants like formic acid. A combination of amine capping ligands, a NS morphology giving a high electrochemically active surface area and synergistic effects between Cu and Pd, resulted in superior catalytic performance for formic acid oxidation compared to other Pd-based catalysts.

Here, we report the one-pot synthesis of PdAu bimetallic NSs using an alkyl quaternary ammonium salt as a soft template. Transmission electron microscopy (TEM) and X-ray photoelectron spectroscopy (XPS) carried out at different reaction times were used to understand the formation mechanism of the NSs and identify changes in the surface chemistry of the NSs. A Suzuki cross coupling reaction was used to assess their catalytic performance under visible light irradiation. Both monometallic Pd and bimetallic PdAu NSs displayed catalytic enhancement with higher yields obtained when the reaction was carried out under visible light compared to conducting the reaction in the dark. The bimetallic NSs also outperformed the monometallic NSs, for example, at a catalyst loading of 0.2 mol % Pd, the PdAu NSs gave full conversion after 3 h at room temperature, while the Pd NSs gave a yield of 69% under the same conditions. The high performance of the surfactant-assisted NSs is in part attributed and the presence of weakly coordinating amine ligands at the surface. Stabilizing ligands such as polymers, small organic molecules, and inorganic species such as Br^–^ ions are commonly used in wet chemical synthesis, yet despite the many literature studies on light-enhanced Suzuki cross coupling, the catalytic role played by the capping ligands has been poorly addressed. To gain insight into ligand-enhanced catalytic and photocatalytic activity, surfactant-assisted NSs were compared with CO-assisted NSs. The surfactant-assisted and CO-assisted NSs displayed a markedly different performance profile for room-temperature cross coupling reactions.

## 2 Experimental Section

### 2.1 Chemicals

Metal precursors palladium(II) chloride (H_2_PdCl_4_) (99.9%), palladium acetylacetonate Pd(acac)_2_ (99%), silver nitrate (AgNO_3_) (99.9%), gold chloride (HAuCl_4_) (99.9%), and tungsten carbonyl W(CO)_6_ were purchased from Sigma-Aldrich. All other synthesis chemicals and solvents were purchased from Sigma-Aldrich except for 1-bromodocosane (95%) which was purchased from ABCR.

### 2.2 Synthesis of Surfactant-Assisted Nanosheets

Surfactant-assisted Pd NSs were synthesized using a modified literature procedure.^27^ Briefly, the long chain alkyl quaternary ammonium surfactant C_22_-QA (Br^–^) was first prepared by the addition of 1.95 g of 1-bromodocosane (10 mmol) and 1.77 mL of trimethylamine (15 mmol) with 75 mL of acetonitrile and refluxed at 95 °C under N_2_ for 22 h. After cooling, the solvent was removed via rotary evaporation. The crude product was washed with diethyl ether three times and dried overnight.

For the synthesis of Pd and PdAu NSs, a 10 mM Pd precursor stock solution was prepared by dissolving 89 mg of PdCl_2_ in 50 mL of a 0.2 M HCl. Pd NSs were synthesized by adding 5 mL of a 0.025 M C_22_-QA (Br^–^) solution in a glass sample vial along with 0.8 mL of 10 mM H_2_PdCl_4_ aqueous solution. The mixture was left to stir until ion exchange had occurred (15 min). One mL of a freshly prepared 0.3 M aqueous solution of l-ascorbic acid was injected slowly into the solution. The reaction solution was placed on an ice bath and left undisturbed at 0 °C with reaction times between 1 and 6 h. Once the reaction had reduced, the product was sonicated with toluene and collected by centrifugation and washed with ethanol. This purification procedure was repeated three times, and the NSs were redispersed in ethanol.

PdAu NSs were synthesized using the same procedure as the Pd NSs but with coaddition of the Pd and Au precursors. For example, PdAu NSs with a molar ratio of 5:1 were prepared by adding 5 mL of a 0.025 M C_22_-QA (Br^–^) solution along with 0.665 mL of a 10 mM H_2_PdCl_4_ and 0.135 mL of 10 mM HAuCl_4_ aqueous solution. PdAu NSs with a molar ratio of 10:1 were synthesized using 0.728 mL of 10 mM H_2_PdCl_4_ and 0.072 mL of 10 mM HAuCl_4_ aqueous solution. After the reaction, the product was sonicated with toluene and collected by centrifugation and washed with ethanol. This purification procedure was repeated three times, and the NSs were redispersed in ethanol.

### 2.3 Synthesis of CO-Assisted Nanosheets

Sixteen mg of Pd(acac)_2_, 30 mg of PVP, 10 mg of citric acid, and 60 mg of cetyltrimethylammonium bromide (98%) CTAB were placed in a test tube vial with 10 mL of DMF, and the solution was bubbled with Ar for 15 min (solution 1). In a separate two-neck round-bottom flask, 100 mg of tungsten hexacarbonyl (97%) W(CO)_6_ was added and purged with Ar. Solution 1 was injected via cannula into the W(CO)_6_ and heated to reflux at 80 °C. The reaction was left to age for 1 h. The product was collected by centrifugation and washed with acetone (4×) to remove excess reagents. The Pd NSs were redispersed in ethanol and used as seeds for the synthesis of PdAg and PdAu NSs. The synthesis was also conducted by changing the polymer ligand from PVP to poly(vinyl alcohol) (PVA) and exchanging the CTAB with other quaternary ammonium salts of different carbon change lengths C8, C12, and C22, i.e., *n*-octyltrimethylammonium bromide (98%), dodecyltrimethylammonium bromide (98%), and *n*-docosyltrimethylammonium bromide, respectively. CO-assisted alloy PdAg and PdAu NSs were synthesized using the same procedure as the Pd NSs, but once the NSs were left to age, a solution containing 15 mg of PVP and 0.692 mL of a 0.025 M AgNO_3_ or 1.66 mL of a 0.01 M AuCl_3_ was added and heated to reflux at 80 °C for a further 1 h. The reaction vessel was wrapped in aluminum foil as AgNO_3_ is photosensitive. The product was collected by centrifugation and washed with acetone (4×) to remove excess reagents and redispersed in ethanol.

### 2.4 Catalytic Evaluation: Suzuki Coupling Reaction with and without Illumination

Suzuki cross-coupling: In a typical experiment 0.026 g of phenylboronic acid (0.22 mmol), 0.046 g of 4-methoxyiodobenzene (0.2 mmol), and 0.0553 g (0.4 mmol) of K_2_CO_3_ were added to 12 mL of ethanol/water (3:1) in a glass vial. The reactions were initiated by the addition of the catalyst (0.05–0.2 mol %) and stirred continuously using a magnetic stirrer. A LED light source was used for reactions carried out under visible light with broad band emission between 400 and 800 nm. The emission spectrum of the LED is given in Supporting Information, see Figure S1. Reactions were carried out in the dark by covering the entire reaction with foil. Reactions were carried out at room temperature (20 °C) or at 80 °C with reaction times between 1 and 6 h. Reactions were carried out in thermostated reactor to ensure temperature regulation, and the reaction temperature was continually monitored. After the reaction was completed, the solution was filtered and washed with DCM (10 mL × 2), and the aqueous layer was removed and washed with DCM (10 mL × 2) and then dried with MgSO_4_. The DCM was removed by rotary evaporation and the product remained. The product was purified before nuclear magnetic resonance (NMR) analysis with deuterated chloroform.

### 2.5 Materials Characterization

XPS was acquired using a KRATOS AXIS 165 monochromatized X-ray photoelectron spectrometer equipped with an Al Kα (*h*ν = 1486.6 eV) X-ray source. Spectra were collected at a takeoff angle of 90°, and all spectra were referenced to the C 1s peak at 284.8 eV. Pd 3d core levels were fit with Shirley backgrounds and Gaussian–Lorentzian profiles. To achieve the best fit, the peak positions were allowed to float with a variable fwhm ranging from 1 to 1.5 for metallic Pd(0) and 1.2 to 1.7 for oxidized Pd components. Peaks shifted to binding energies (BEs) greater than +1.5 eV of the elemental Pd(0) peak were assigned to bulk oxide phases PdO. TEM analysis was performed using a JEOL 2100 electron microscope at an operating voltage of 200 kV, and high-resolution TEM and energy-dispersive X-ray (EDX) analyses were carried out on an FEI Titan TEM, at an operating voltage of 300 kV. Scanning electron microscopy (SEM) was carried out on a FEI Helios NanoLab 600i operating at 30 kV and 0.69 nA, with an attached EDX Oxford X-Max 80 detector. X-ray diffraction (XRD) was carried out using a Philips X’pert Pro MPD, equipped with a Panalytical Empyrean Cu X-ray tube and a Philips X’celerator detector. Fourier transform infrared (FTIR) spectra were recorded on a PerkinElmer Spectrum Two FTIR spectrometer operating in the range of 4000–450 cm^–1^ with a resolution of 4 cm^–1^ and spectra were averaged from 20 scans. NMR samples were run in deuterated chloroform (CDCl_3_). ^1^H NMR spectra were recorded on Bruker Avance III 300 NMR spectrometers, in proton-coupled mode using tetramethysilane as the internal standard.

## 3 Results and Discussion

### 3.1 Synthesis of Alloy Surfactant Assistant Nanosheets

**Scheme 1:**
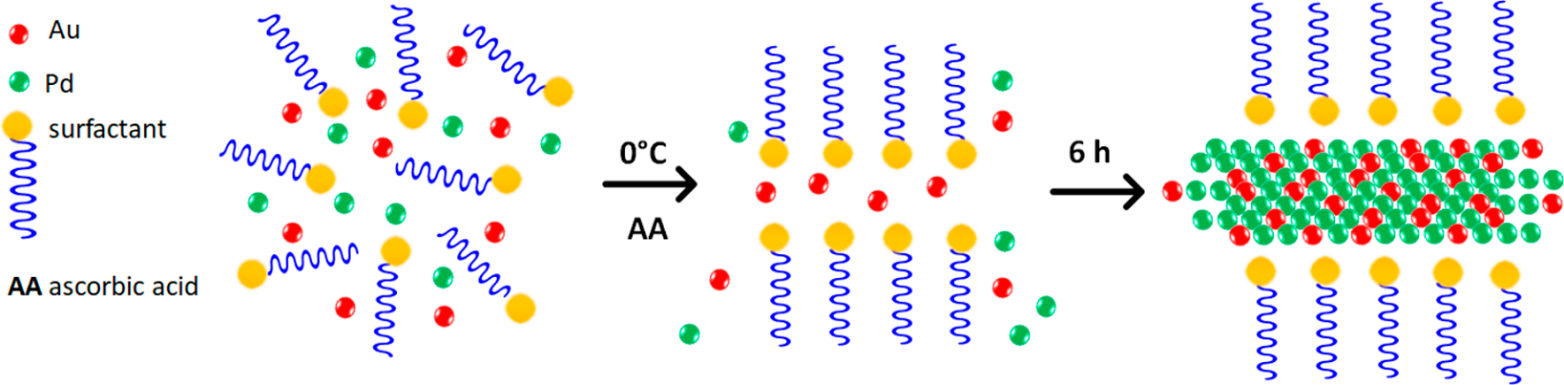
Surfactant-Assisted Synthesis of PdAu NSs

**Figure 1:**
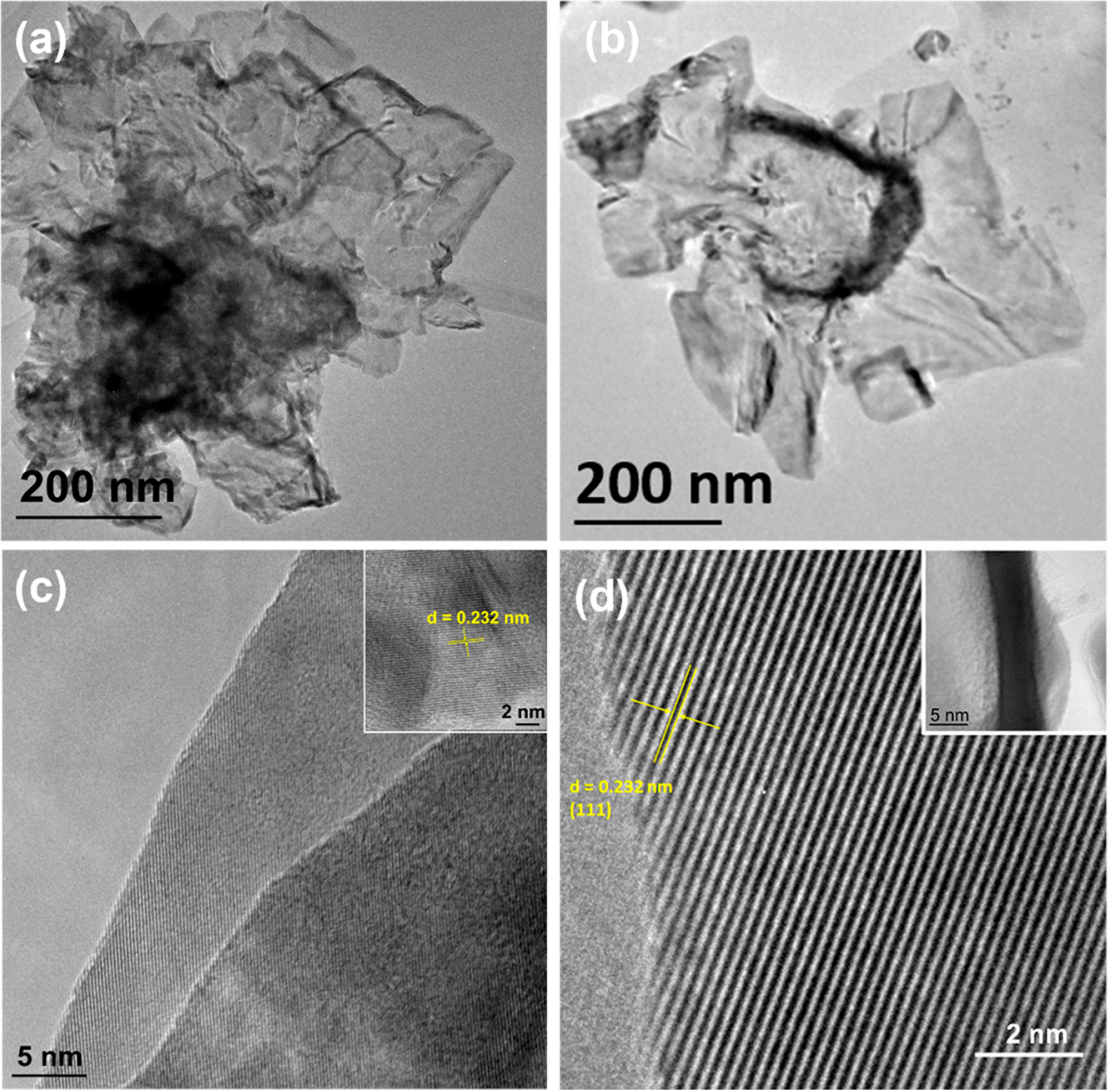
TEM images of (a) PdAu 5:1 NSs, (b) PdAu 10:1 NSs and (c) and (d) HRTEM with lattice planes of a PdAu 5:1 NS.

Bimetallic 2D NSs with extended sheet lengths were synthesized by reduction of Pd and Au precursors in the presence of long chain alkyl quaternary ammonium surfactant templates, as illustrated in Scheme 1. Figure 1a shows a TEM of NSs formed using a Pd:Au molar ratio of 5:1, and Figure 1b shows NSs produced using a molar ratio PdAu 10:1 NS, which have an irregular morphology. The TEM image shown in Figure 1c displays two overlapping sheets, with the inset figure showing the NSs to be crystalline. Figure 1d shows a high-resolution image of a NS with a lattice spacing of 0.23 nm, which is close to that of (111) face centered cubic Pd, although the *d*-spacings of individual Au and Pd are quite close, i.e., 0.236 and 0.225 nm, respectively.^28^ The TEM image also shows a high density of atomic steps along the edge of the NS. The mean thickness of the NS estimated by TEM, as shown in the inset of Figure 1d, was ca. 5 nm, equivalent to over 20 atomic layers thick, which is greater than the previously reported thickness of surfactant template monometallic Pd NSs, ca. 2.5 nm.^27^

**Figure 2:**
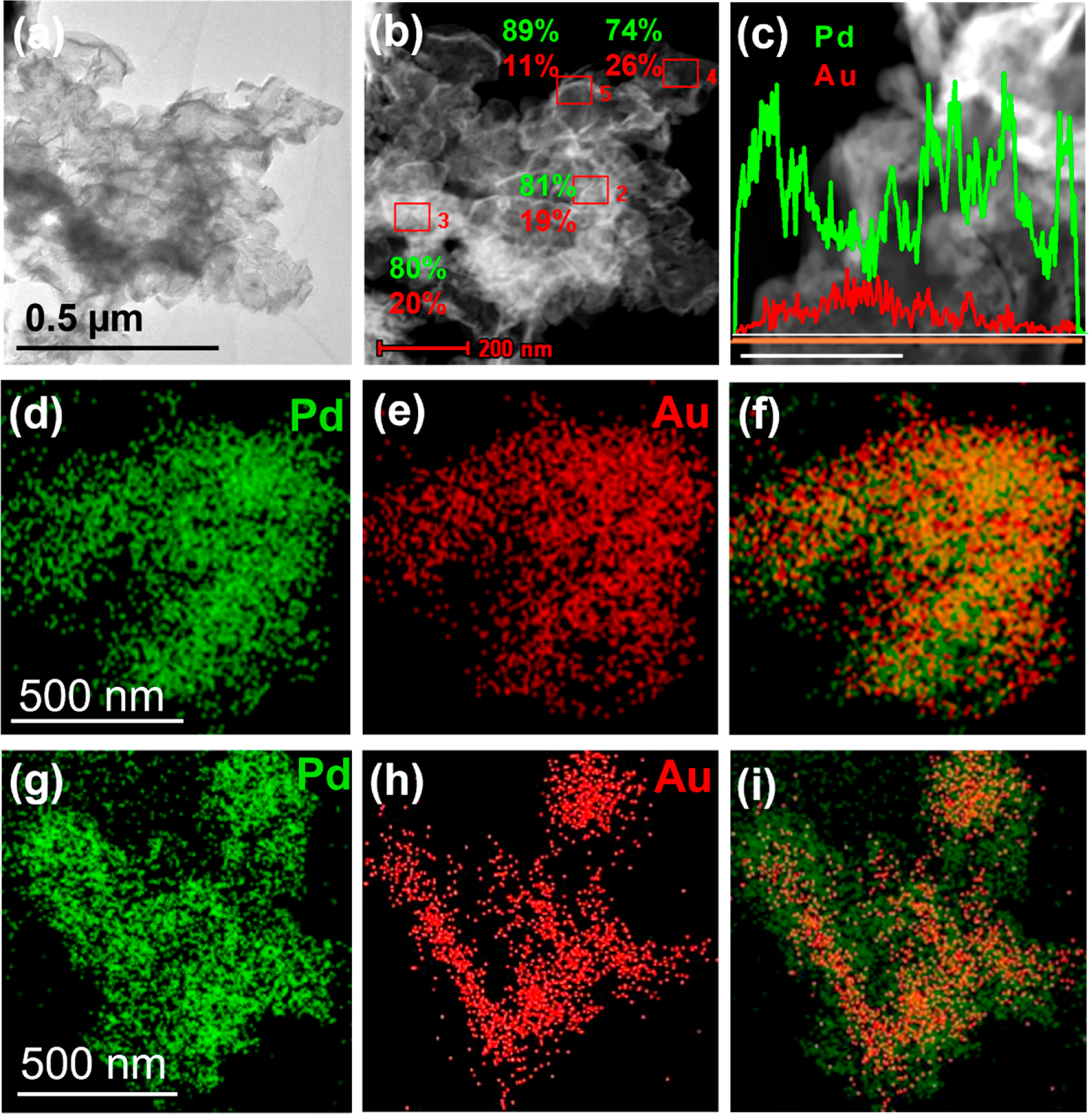
(a) TEM image of PdAu NSs 5:1. (b) STEM image and corresponding EDX quantification of atomic concentrations. (c) EDX line map of PdAu 5:1 NSs (scale bar is 100 nm). (d–f) Pd, Au, and overlaid EDX maps for PdAu 5:1. (g–i) Pd, Au, and overlaid EDX maps for PdAu 10:1 NSs.

Elemental analysis was used to assess the distribution of Au and Pd in the NSs. Figure 2a,b shows a low-resolution TEM image and corresponding STEM image with EDX point analysis, respectively. The Pd:Au quantification data given in Figure 2b were consistent across different areas and also in good agreement with the Au and Pd precursor molar ratio used for the synthesis (5:1). Figure 2c displays an EDX line scan across a PdAu NS and confirms the presence of both Au and Pd. Figure 2d–f shows the Pd, Au, and the overlaid EDX maps for the PdAu 5:1 NS, and Figure 2g–i shows the Pd, Au, and overlaid EDX maps for the 10:1 NS, respectively. The individual elemental EDX maps show a uniform dispersion of Au and Pd; however, closer evaluation of the overlaid maps reveals the presence of Pd-rich regions along the edges. Noticeably in Figure 2h, which corresponds to the PdAu 10:1 NSs, the Au concentration along the edges is clearly lower than the central portion. Additional EDX maps illustrating this pattern of dispersion are shown in the Supporting Information (see Figure S2). The presence of PdAu alloy and Pd-rich domains is also indicated by the XRD analysis. The XRD pattern shown in Supporting Information Figure S3 further supports the formation of crystalline NSs, with the characteristic peaks of Pd (111) and (200) observed at 2θ values of 40.1° and 46.6°, respectively, for the monometallic Pd NSs.^29^ The XRD pattern of the PdAu NSs shows a Pd (111) peak downshifted to 39.8°, which was broader (fwhm = 0.46) compared to monometallic Pd NSs (fwhm = 0.32), typical of a PdAu alloy. An additional shoulder was also observed at 38.4°, attributed to the Au (111) peak but upshifted from the standard Au 111 diffraction peak.^30^ A truly homogeneous alloy would be expected to display a single 111 diffraction peak for Au and Pd, and the XRD analysis supports the EDX analysis indicating the presence of two alloy domains.

**Figure 3:**
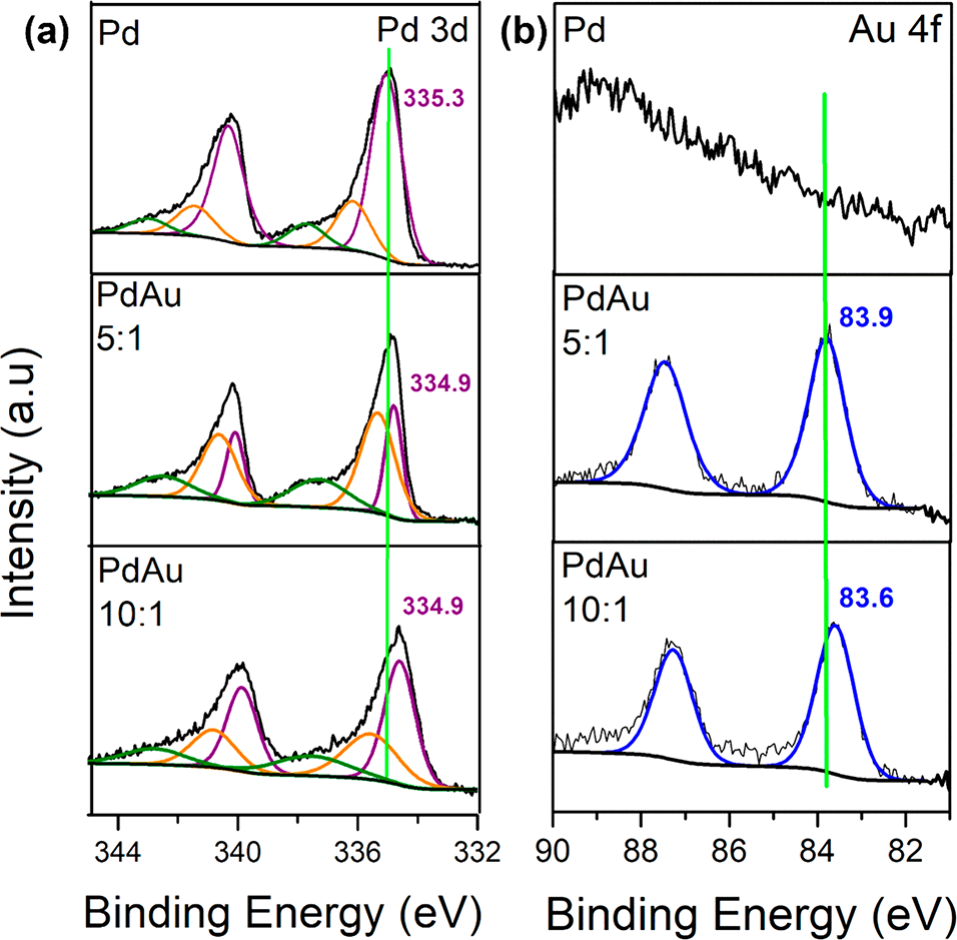
XPS analysis of (a) Pd 3d and (b) Au 4f core levels of Pd, PdAu 5:1 and 10:1 NS.

XPS analysis was carried out to provide further insight into the modified electronic properties of the bimetallic NSs. Figure 3a compares the Pd 3d core level spectra of Pd, PdAu 10:1, and PdAu 5:1 NSs. The Pd 3d_5/2_ core level of the monometallic Pd NSs was centered at a BE of 335.3 eV, consistent with Pd(0). Minor oxide contributions were observed at 336.4 eV, corresponding to PdO and surface oxide species and a smaller shoulder peak at 337.8 eV, which is assigned to PdO_2_.^29^ The BE of Pd 3d was negatively shifted from 335.3 eV in the Pd NSs to 334.9 eV in the PdAu NSs. These BE shifts indicate a change in the electronic structure and *d*-band modification of Pd, due to charge transfer between Au and Pd when they are alloyed. Peaks shifts were also observed in the Au 4f core level, shown in Figure 3b. No Au was detected in the Pd NSs, as expected. The BE for Au(0) is usually reported at 84 eV,^31^ and the Au 4f_7/2_ peak of the Pd:Au 5:1 was centered at 83.9 eV, while the Pd:Au 10:1 peak was centered at 83.6 eV. This negative peak shift in the Au 4f BE can be attributed to the electronic modification of Au species by Pd.

**Figure 4:**
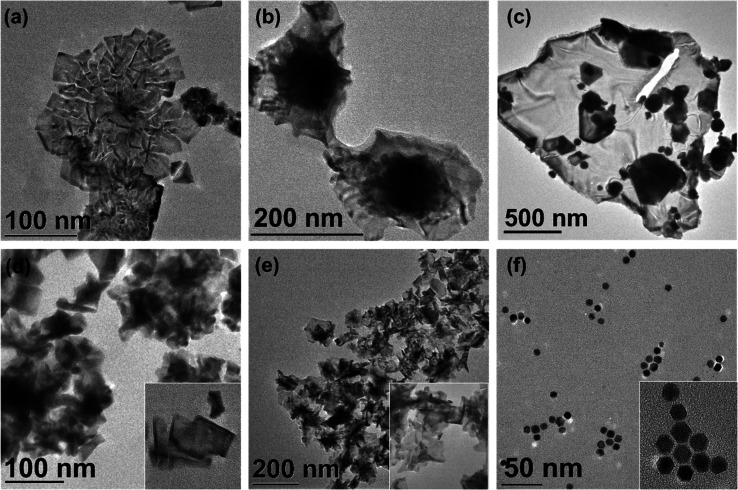
TEM images of nanostructures obtained with surfactant concentration of 0.025 M and Pd:Au molar ratio of (a) 5:1 (b) 2:1 and (c) 1:5. (d) PdAu nanostructures synthesized using C_22_N-COOH (Br^–^) surfactant, (e) Pd NSs synthesized using C_22_-COOH (Br^–^), and (f) cuboctahedral NP formed using a C_22_-Py (Br^–^) surfactant.

A number of reaction parameters such as the surfactant ligand, surfactant concentration, Au:Pd molar ratio, ascorbic acid concentration, and temperature were altered to determine their effect on NS formation, which are summarized in Table S1 in Supporting Information. As outlined in Table S1, the optimal conditions for NS formation were using a C_22_-QA surfactant at a concentration of 0.025 M, a surfactant:Pd molar ratio of 1:0.025, reaction temperature of 0 °C and 6 h. Under these conditions, tuning the Pd:Au molar ratio produces PdAu 10:1 or 5:1 NSs, as previously shown in Figures 1 and 2. Increasing the Au concentration further using a Pd:Au molar ratio of 2:1 gave a mixture of poorly defined NSs and NPs due to heterogeneous nucleation of the Au precursor, as shown in Figure S4 in the Supporting Information. Increasing the molar ratio of the Pd precursor relative to surfactant concentration (1:0.25) produced NSs, but competitive growth of NPs was also evident as shown in Figure 4a–c. The higher surfactant concentration is required for surfactant templated growth of NSs and minimizing the growth of NPs. Temperature was also a critical factor in the synthesis, with NSs only forming at an aging temperature of 0 °C, and reactions at a higher temperature produced only irregular NPs. The headgroup of the surfactant also played a critical role in the synthesis. Xu et al.^27^ reported that long chain alkyl ammonium salts with alkyl groups, carboxylates, and pyridyl head groups all served as templates for Pd NSs, but this was not observed for the PdAu NSs, where the alkyl quaternary ammonium salt was the only capping agent successful in achieving a NS morphology. Substituting the methyl group on the ammonium salt for a carboxylic acid formed irregular nanostructures, as shown in the TEM analysis, see Figure 4d. While the carboxylic acid surfactant was not successful for the synthesis PdAu NSs, it was effective for making monometallic Pd NS (Figure 4e), which may be attributed to the low coordinating ability of carboxylic acid groups toward Au. When the alkyl ammonium salt terminated with a pyridyl group was used, cuboctahedral nanocrystals were formed, as shown in Figure 4f.

### 3.2 Growth Mechanism of PdAu Alloy Nanosheets

**Figure 5:**
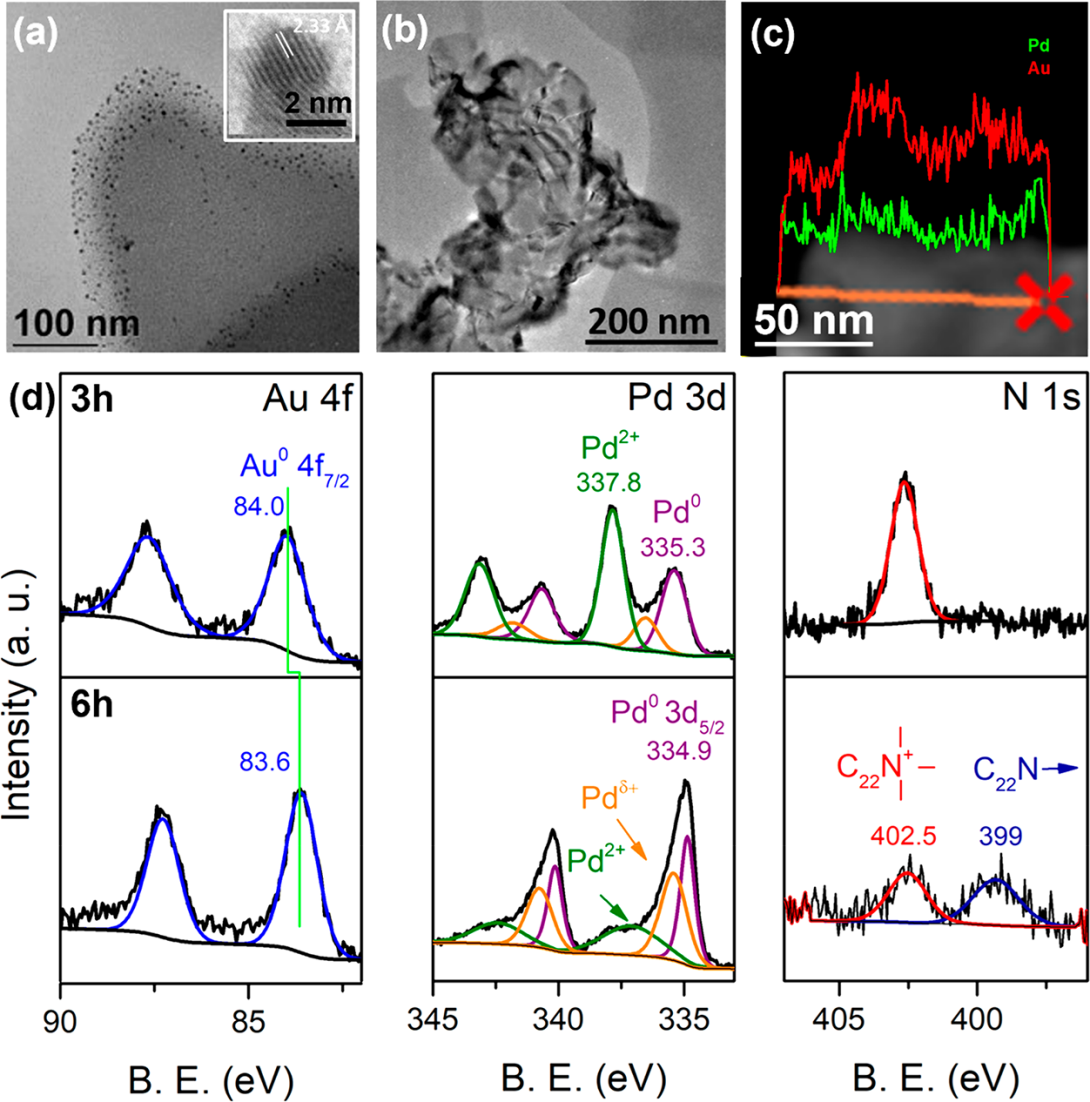
(a) TEM image 1 h after reaction showing NP formation. (b) TEM image from an incomplete reaction (3 h) showing formation of NSs. (c) EDX line map of NS after 3 h. (d) Au 4f, Pd 3d, and N 1s XPS core levels from incomplete and complete NS reactions.

To understand the formation mechanism of the PdAu NSs, aliquots were taken from reaction solutions at various times and analyzed by TEM. The TEM analysis was further correlated with XPS and EDX analyses of uncompleted reactions to determine changes in the chemical state and composition of the NSs. Figure 5a displays a TEM image 1 h after the reaction, using a Pd:Au molar ratio of 5:1, which shows the presence of small diameter NPs (∼2 nm) dispersed in a carbon matrix consisting of the long chain alkyl ammonium salt. HRTEM of the NPs shown in the Figure 5a inset shows an interplanar distance of 0.233 nm, which in is good agreement with the face centered cubic structure of Au (111).^32^ As the reaction proceeds, the formation of NSs is observed at 3 h, as shown in Figure 5b. EDX analysis of a NSs isolated from the incomplete reaction showed the presence of a Au-rich NSs, as shown in EDX the line scan in Figure 5c. Correlating the TEM and EDX analyses with XPS analysis, (Figure 5d), this incomplete reaction mixture confirmed the presence of metallic Au, as shown in the Au 4f spectra with the BE of 84 eV, consistent with Au^0^ and indicating reduction of the Au precursor. The Pd 3d core level shows the presence of both metallic Pd^0^ centered at 335.3 eV and Pd^2+^ centered at 337.8 eV, indicating partial reduction of the Pd precursor at this stage of the reaction. Analysis of the peak areas in the deconvoluted Pd 3d_5/2_ core level determined that ∼35% of the Pd precursor had been reduced to Pd^0^. In addition to Pd^0^ and Pd^2+^ species, there was a small peak at a BE of 336.5 eV, typically associated with electron-deficient Pd species (Pd^δ+^) such as a surface oxide.^33^ XPS analysis of NSs from the completed reaction (6 h) is also displayed in Figure 5(d). The Au 4f BE of the NSs after 6 h was downshifted with the Au 4f_7/2_ peak now centered at 83.6. eV. The corresponding Pd 3d core level of the NSs after 6 h showed primarily Pd^0^, due to reduction of the remaining precursor and the Pd 3d_5/2_ peak position after 6 h was also downshifted to 334.9 eV, compared to the NS analyzed at 3 h. This downshift is likely attributed to increased electron density of Pd associated with alloying. Negative shifts of both the Au 4f and Pd 3d core level bands in bimetallic species have been reported.^34^

Based on the ratio of the Au 4f to Pd 3d peaks, XPS analysis determined a Pd:Au ratio of 9.4:1 in the uncompleted reaction and 9:1 for the completed NS reaction, which is in excellent agreement with the starting molar ratio of the Pd and Au precursors (10:1). These studies imply that the formation mechanism of the PdAu NS is initiated by the formation of small diameter Au NP seeds dispersed in the long chain alkyl quaternary ammonium salt surfactant which serve as a template for the growth of NSs. The faster reduction of Au is understandable given the different standard reduction potential of Au and Pd ions (AuCl_4_^–^/Au, 1.002 V; PdCl_4_^2–^/Pd, 0.591 V).^35,36^ Au-rich alloy NSs are initially formed and the Pd concentration increases with reduction of the precursor as the reaction proceeds. This mechanism accounts for the observed EDX mapping analysis showing the Pd-rich regions located around the NS edges, as after the Au precursor has been consumed, continued growth of the NS produces Pd-rich domains as identified by EDX.

The evolution of changes to the NS surface chemistry during growth was also evaluated by assessing the N 1s core level which is shown in Figure 5d. The N 1s core level of the incomplete reaction mixture showed a single peak at a BE of 402.5 eV, which is characteristic of the quaternary ammonium salt.^37^ The N 1s of the NSs formed showed the presence of two peaks, one centered at 402.5 eV due to the ammonium salt and a second peak at a lower BE at 399 eV, which is characteristic of amine species or metal bound amines.^38^ There are approximately equal amounts of these two N environments (52% and 48% attributed to species at a BE of 399 and 402.5 eV, respectively) based on the deconvoluted N 1s spectrum. As the reaction proceeds, there is also significant reduction in the Br^–^ ion species (see Supporting Information Figure S5). Based on the N 1s and Br 3d signal, the N:Br ratio in the incomplete reaction is 1:2.3, which falls to 1:0.54 in the NSs formed, suggesting that the surface of the NSs are passivated with a mixture of the noncoordinated quaternary ammonium salts (C_22_-N^+^Br^–^) and a more electron-rich N functionality. Quaternary ammonium salts undergo C–N cleavage to form amines in the presence of many metals, which may explain the presence of the peak at 399 eV.^39^ FTIR analyses of the surfactant and the NSs are shown in the Supporting Information Figure S6 and display significant peak broadening of the C–N vibration (950–1100 cm^–1^) for the Pd and PdAu NSs compared to the bulk quaternary ammonium salt.

### 3.3 Catalytic Evaluation of Nanosheets

The catalytic performance of the NSs was assessed in the Suzuki cross-coupling reaction of 4-iodoanisole (4-methoxyiodobenzene) and phenylboronic to give 4-methoxybiphenyl. Table S2 in the Supporting Information summarizes some literature reports of Suzuki cross coupling reactions under conventional heating and light-driven catalysts.^40−49^

**Figure 6:**
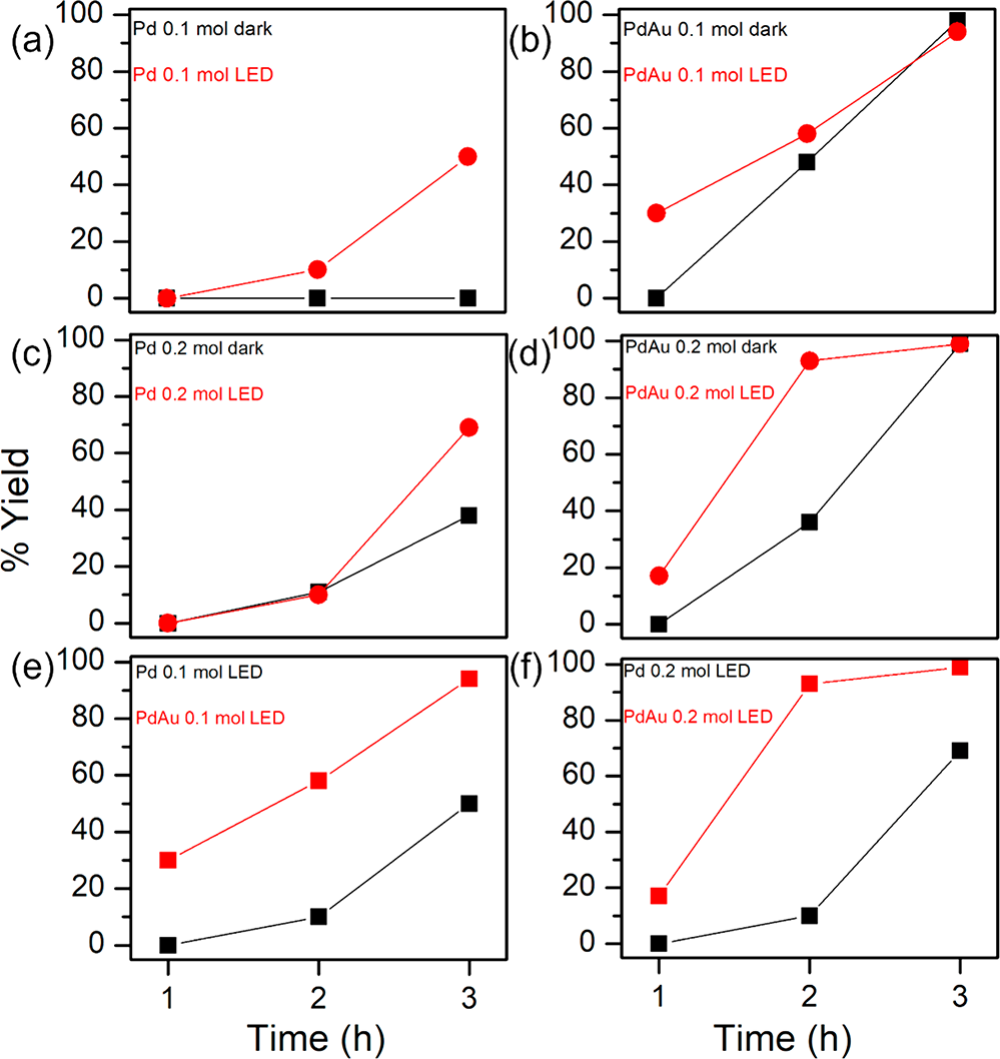
(a–f) Catalytic evaluation plots of the cross-coupling Suzuki reaction catalyzed using Pd and PdAu NSs at 0.1 and 0.2 mol % under visible light irradiation and in the dark.

The photocatalytic enhancement was evaluated by conducting reactions under visible light irradiation using an LED light source and in the dark (see Experimental Section for further details). NMR analysis confirmed no homocoupling of the boronic acid occurred, and the cross coupled biphenyl was the only product formed in the reaction (see Supporting Information Figure S7). The effects of catalyst concentration, temperature, and reaction time were also evaluated. To investigate the effect of the NS surface chemistry, NSs prepared via a CO-assisted route were also synthesized, and their catalytic performance was studied as a comparison to the surfactant-assisted NSs. The catalytic activity of the Pd and PdAu (5:1) NSs in the Suzuki cross-coupling reaction shows two distinct enhancement effects, one attributed to the alloy effect and the other to a photocatalytic enhancement. Figure 6a,b shows reaction yields obtained for Pd and PdAu NSs, respectively, under light illumination and in the dark, at reaction times of 1, 2, and 3 h. The Pd catalyst concentration in both reactions was 0.1 mol %, i.e., the Pd concentration was equivalent in the reactions catalyzed by monometallic and bimetallic NSs. Both Pd and PdAu NSs showed improved yields under light illumination. After 3 h, the yield obtained for the Pd NSs increased from 0 to 51% under light illumination, while the PdAu NSs increased from 30% (1 h) to 98% (3 h). One notable difference between the Pd and PdAu NSs was the longer induction period displayed by the Pd catalysts compared to the bimetallic catalyst. Induction periods are commonly observed with NP-catalyzed Suzuki reactions and often attributed to the formation of active Pd species that are generated from the surface.^50^ After 1 h at a 0.1 mol % Pd loading, no product was formed using Pd NSs, compared to a 30% yield after 1 h for the PdAu NSs. The higher performance of Pd alloys toward Suzuki coupling has been reported in numerous alloys and may be attributed to the increased electron density of the Pd, which correlates with XPS analysis, indicating the Pd is more electron rich in the bimetallic NSs.^51,52^ The increased electron density of the Pd facilitates oxidative addition of the aryl halide.^53^

NP-catalyzed Suzuki reactions are known to depend on catalyst concentration. Increasing the catalyst concentration can sometimes decrease the activity due to catalyst aggregation. The reaction was evaluated at catalyst concentrations of 0.1 and 0.2 mol %. The same reactivity trend was observed using 0.2 mol % loading, with the bimetallic NSs outperforming the monometallic Pd NSs, and yields were greater under visible light irradiation compared to reactions carried out in the dark, as shown in Figure 6c,d. The higher catalyst loading improved yields generally, e.g., Pd NSs showed no activity in the dark at 0.1 mol %, but this increased to 38% at 0.2 mol %. For the PdAu NSs, there was an even larger yield improvement under light irradiation, achieving a 93% yield with a Pd catalyst loading of 0.2 mol % after a 2 h reaction time. A yield of 93% after 2 h for a Suzuki reaction carried out at room temperature is significant. Turnover numbers (TONs) and turnover frequencies (TOFs) for the reactions are tabulated in Figure S3 (Supporting Information).

Figure 6e,f highlights the effect of using a bimetallic catalyst compared to a monometallic catalyst. The PdAu NSs display better photocatalytic enhancement compared to the monometallic NSs having higher yields at all reaction times. Even in the absence of light, the PdAu NSs produce higher yields than that of the Pd NSs, indicating the presence of the alloy enhancement effect. XPS analysis revealed that the surface is electron rich in the PdAu NSs, which is known to favor the oxidative addition of the aryl halide, and this may account for the higher activity of the PdAu NSs even in the absence of light. The combined catalytic enhancement effect when using light and a bimetallic catalyst is significant; at a Pd concentration of 0.1 mol % using monometallic Pd NSs in the dark, no product was formed after 3 h. Keeping the same Pd concentration, but using the PdAu NSs and under visible light illumination, the reaction goes to completion in the same time period. Commercial Pd on carbon was also evaluated as a reference catalyst to compare performance improvement when using a NS morphology. At a catalyst concentration of 0.2 mol %, the yield obtained at room temperature was only 16% after 3 h with commercial Pd/C.

Catalysis by plasmonic nanostructures can be attributed to hot carriers or photothermal effects, and both have been observed in Suzuki cross-coupling reactions.^16,54^ Teranishi and co-workers showed that plasmon-enhanced catalytic activity of Pd hexagonal nanoplates was dominantly driven by hot electron injection.^49^ In AuPd nanostructures, photothermal effects can be significant and give rise to considerable increases in the solution temperature, e.g., Buskens et al.^55^ reported Pd NP tipped Au nanorods increased the solution temperature from 25 to 60 °C under illumination, but the large heating effect was attributed to the photothermal heat generation by the Au nanorod. To assess photothermal effects in the NSs, the temperature of the reaction solvent (EtOH:water) in the presence of 0.2 mol % catalyst was monitored over a 3 h period. A small increase of 3 °C (average of 3 runs) was observed for AuPd NSs, while a negligible increase was observed for the Pd NSs. The minimal heating effect is attributed to the low absorption cross section of the NSs and the use of LED illumination.^56^ While a photothermal contribution cannot be ruled out, as the bulk temperature of the reaction solution may differ substantially from the local surface temperature of the NSs, hot electrons that undergo oxidative addition with the aryl halide rather than a photothermal effect are likely the dominant mechanism of the photocatalyzed Suzuki reaction.

**Figure 7:**
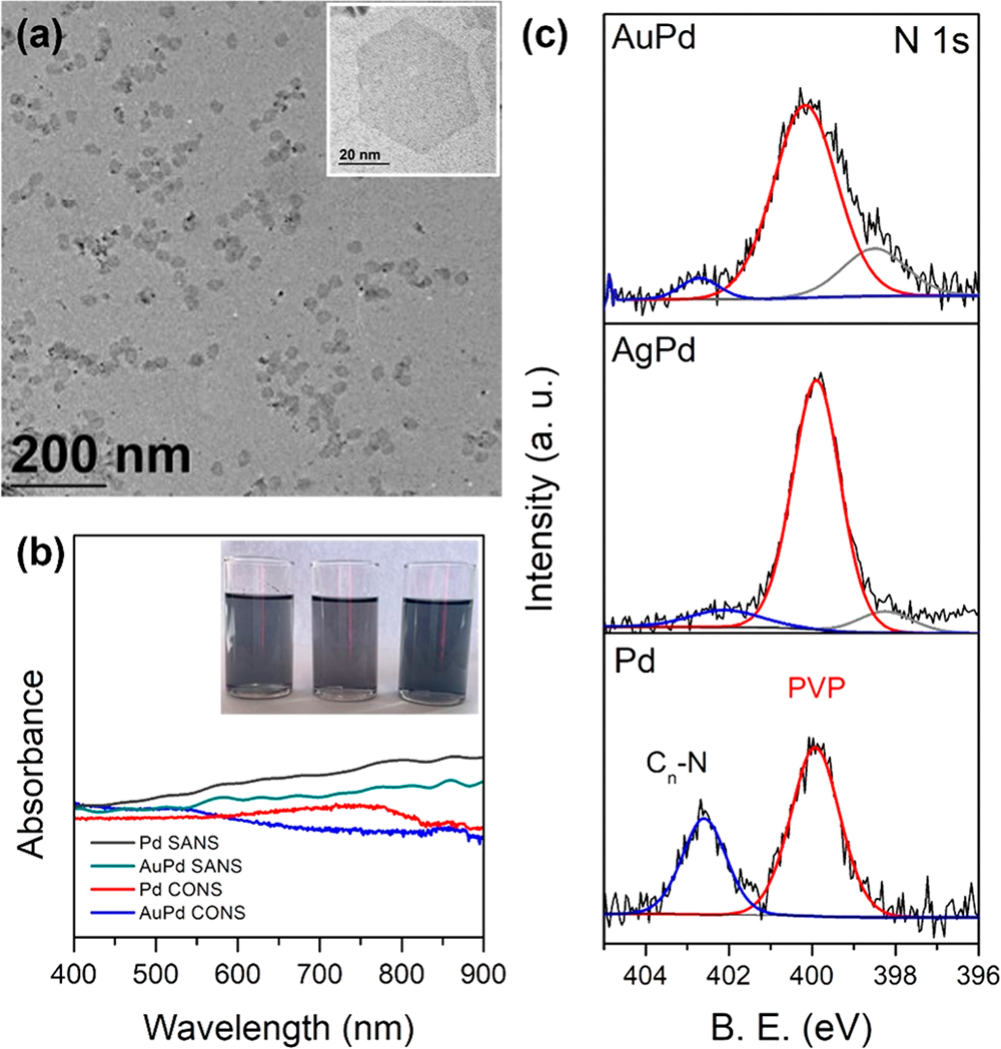
(a) Low-resolution TEM image. (b) UV–vis spectra of surfactant-assisted and CO-assisted NSs. (c) N 1s core level of Pd, PdAg and PdAu CO-assisted NSs.

The high catalytic performance of the NSs can also be attributed to their 2D morphology, with a high density of surface atoms. The presence of weakly coordinating ammonium salts should play a role in influencing the catalyst activity. To further evaluate the role of capping ligands on the catalytic behavior of the NSs in the Suzuki cross coupling reaction, CO-assisted Pd NSs alloyed with traditional plasmonic metals were synthesized. The strong adsorption of CO onto Pd (111) facets produces well-defined hexagonal NSs, as showed in the low-resolution TEM image in Figure 7a. In addition to CO, organic stabilizing ligands PVP and CTAB are also required to prevent aggregation of the NSs (see Experimental Section for further details). The UV–vis spectra of the surfactant-assisted and CO-assisted NSs are shown in Figure 7b. The surfactant-assisted NSs show increasing absorption across the visible spectrum (400–900 nm). The inset in Figure 7b shows the image of a NS solution that has a characteristic blue appearance. The Pd and AuPd and spectra are similar and are attributed to the relatively low Au concentration in the NSs.^57^ Furthermore, the absence of a peak observed 530 nm, i.e., the LSPR for Au NPs further indicates that Au is present within the NSs with very few spherical Au NPs in the suspension.^58^ In contrast, the PdAu CO-assisted NSs display an increase in absorption around 500 nm associated with the presence of the Au NPs that formed from heterogeneous nucleation of the Au precursor. TEM analysis confirmed that the synthesis of PdAu NS using a CO-assisted method produced a mixture of NSs and NPs, due to heterogeneous nucleation of the Au (see Supporting Information, Figure S8). The surface chemistry of the CO-assisted NS was investigated by XPS. Figure 7c shows the N 1s core level of the Pd, PdAg, and PdAu NSs. The Pd NSs showed the presence of two N peaks, one at 398.8 eV attributed to the PVP ligands and another at 402.6 eV attributed to the quaternary ammonium salt surfactant.^59^ The PdAu and PdAg NSs displayed primarily one peak associated with the PVP ligands, with minor peaks associated with the ammonium surfactant.

**Figure 8:**
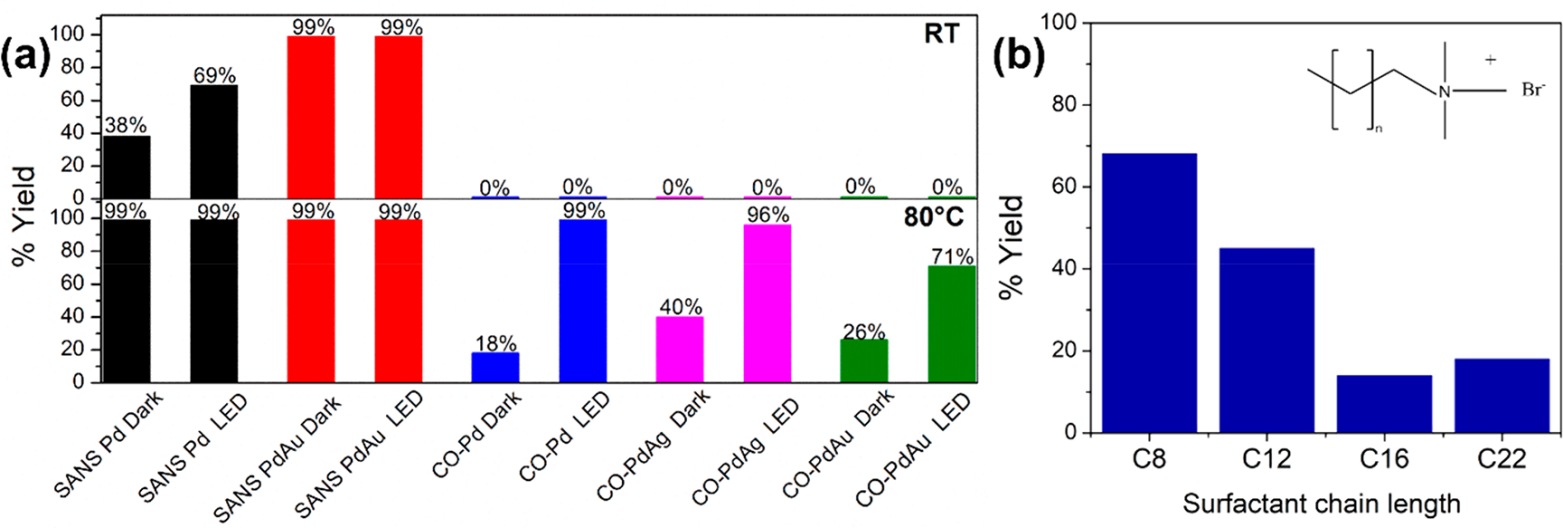
(a) Reaction yields obtained from surfactant-assisted NSs (SANS) and CO-assisted NSs (CO-NS) after 3 h with 0.2 mol % Pd at room temperature (RT) and at a temperature of 80 °C. Note that the PdAu CO-assisted NSs is comprised of a mixture of Au NPs and NSs. (b) Reaction yields of biphenyl product obtained from CO-assisted Pd NSs synthesized using alkyl quaternary ammonium salts with different chain lengths (C8, C16, C12, and C22).

Figure 8a compares the reaction yields obtained for surfactant-assisted NSs and CO-assisted NSs in the Suzuki cross coupling reaction, carried out at 0.2 mol % in the dark and under light irradiation at room temperature and at 80 °C. In contrast to the excellent activity observed for the surfactant-assisted NSs, the CO-assisted NSs showed no activity at room temperature, and there was no impact of light illumination for reactions carried out at room temperature when catalyzed by Pd, PdAg, or PdAu. A catalyst concentration of at least 3 mol % was required before any product was formed at room temperature. While the surfactant-assisted and CO-assisted NSs have structural differences, e.g., the surfactant-assisted NSs are thicker and larger (∼100 nm) compared to the CO-assisted NSs (45 nm),^23,27^ the poor catalytic activity of the CO-assisted NS is primarily attributed to the heavily passivated surface due to the presence of CO, PVP, and CTAB. The reaction temperature was increased to 80 °C to promote remote removal of physisorbed surface ligands.^50^ The elevated reaction temperature resulted in higher yields for monometallic and alloy NSs, as shown in Figure 8a. The yield for reactions crried out in the dark remained poor, at only 18%, but there was a significant photocatalytic enhancement in the presence of light, with a yield up to 99% obtained. A similar photocatalytic enhancement was observed for the CO-assisted PdAg NSs, with the yield increasing to 40% in the dark to 96% under light. Although the PdAu CO-assisted NSs contained a mixture of sheets and Au NPs, a higher yield was obtained under illumination (71%) compared to in the dark (26%), due to the presence of a discrete plasmonic component (Au NPs) and a catalytic component (Pd NSs). As observed with the surfactant-assisted NSs, the bimetallic CO-assisted NSs showed superior performance to the monometallic Pd NS, both the absence and presence of light irradiation attributed to the synergistic alloy effect.

Efforts to partially remove surface ligands, which would be advantageous for catalysis, proved challenging. Solvent washing was ineffective, while chemical cleaning using NaBH_4_ led to dissolution of the NSs as confirmed by TEM analysis (see Supporting Information Figure S10). By alternating the capping ligands used in the synthesis reaction, it was found that the presence of CO, PVP, and a long chain alkyl ammonium salt, e.g., CTAB, were all essential for successful NS formation. When the PVP was substituted for other polymers, such as PVA, no NSs formed due to the insufficient stabilizing ability of the polymer. When the alkyl quaternary ammonium salt was removed from the synthesis, NSs did not form; however, ammonium salts with different alkyl chain lengths could be exchanged. Changing the surfactant chain length did not impact on the 2D morphology of the NSs, but there was as an increase in their mean size when synthesized using a shorter chain length surfactant. As shown in the size distribution analysis in Figure S11 (Supporting Information), the mean widths for the NSs using C22, C16, C12, and C8 were 29 nm, 22 nm, 43 nm, and 57 nm, respectively. Figure 8b shows the yield of the biphenyl after 3 h at 80 °C, using CO-assisted NSs prepared with different alkyl chain lengths. The product yield decreased with increasing chain length going from 68% for C8 to 18% for C22. While it is difficult to disentangle a potential size effect on catalytic behavior, the chain length of the ammonium salt surfactant clearly has an impact on the catalytic behavior. The higher performance of the shorter chain length is most likely attributed to the steric effect associated with a longer alkyl chain length impeding access to the surface of the NSs.^60^ This study further highlights the importance of considering the impact of the capping ligands on the catalytic performance of the nanostructures with similar morphology.

## 4 Conclusions

We report a one-pot synthesis of surfactant-templated PdAu NSs. TEM, EDX, and XPS analyses indicate that the growth mechanism of the NSs is initiated through the formation of Au NP seeds dispersed within the quaternary ammonium salt surfactant. These NP seeds grow into 2D PdAu NSs, and due to fast reduction of the Au precursor, the edges of the NSs are Pd rich as demonstrated by EDX. The catalytic and photocatalytic activity of the Pd and PdAu NSs were evaluated in room-temperature Suzuki coupling reactions under light and dark conditions. A significant increase in product yields was observed under visible light illumination, and the PdAu NSs showed the greatest photocatalytic enhancement compared to Pd NSs. The catalytic activity is attributed to the 2D morphology of the NSs, the electron-rich Pd surface, and the presence of weakly bound capping surface ligands. We further synthesized CO-assisted NSs to gain insight into the impact of surface chemistry using catalysts with a similar 2D morphology. A comparative study shows that the catalyst surface chemistry has a significant impact on both the catalytic and photocatalytic behavior of ligand-stabilized NSs, with the heavily passivated CO-assisted NSs having poor reactivity. Both the surfactant-assisted and CO-assisted NSs showed the same trend with the bimetallic NSs displaying greater lighter enhancement compared to monometallic Pd NS. This work highlights the importance of considering the catalyst surface chemistry in addition to catalyst morphology for light-enhanced reactions.

## Supporting Information Available

Additional analysis and results, including TEM, XRD, EDX, FTIR, and XPS survey scans of the surfactant-assisted NSs, TEM and size distribution profiles of the CO-assisted NSs, NMR spectra of the product of the Suzuki reaction, table of reaction parameters, literature results, and TOF of each catalyst (PDF)
