# Supplementary material for: PdAu Nanosheets for Visible-Light-Driven Suzuki Cross-Coupling Reactions
Source: ACS Appl Nano Mater. 2022 Oct 24;5(11):16196–206. doi: 10.1021/acsanm.2c03216 (PMC9706499; doi:10.1021/acsanm.2c03216)
Supplement: Supplementary file 1 — an2c03216_si_001.pdf [file an2c03216_si_001.pdf]

Supporting Information

PdAu Nanosheets for Visible Light Driven Suzuki Cross-Coupling Reactions

Éadaoin Casey, Prof. Justin. D Holmes, Dr. Gillian Collins*

[1] School of Chemistry, University College Cork, Cork, T12 YN60, Ireland.

[2] AMBER Centre, Environmental Research Institute, University College Cork, Cork, T23 XE10, Ireland.

Corresponding Author Information

*To whom correspondence should be addressed: Tel: +353 (0)21 4205143. E-mail: g.collins@ucc.ie

Orcid iD: 0000-0002-5950-2457

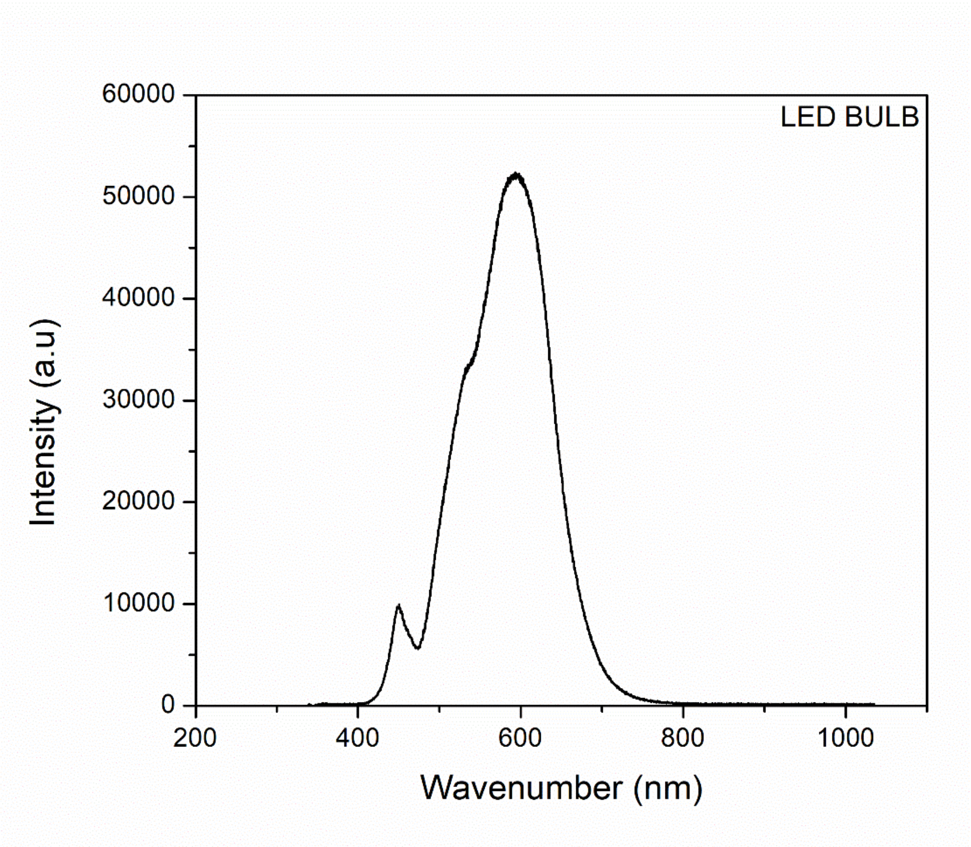

Figure S1: Emission Spectra of LED light

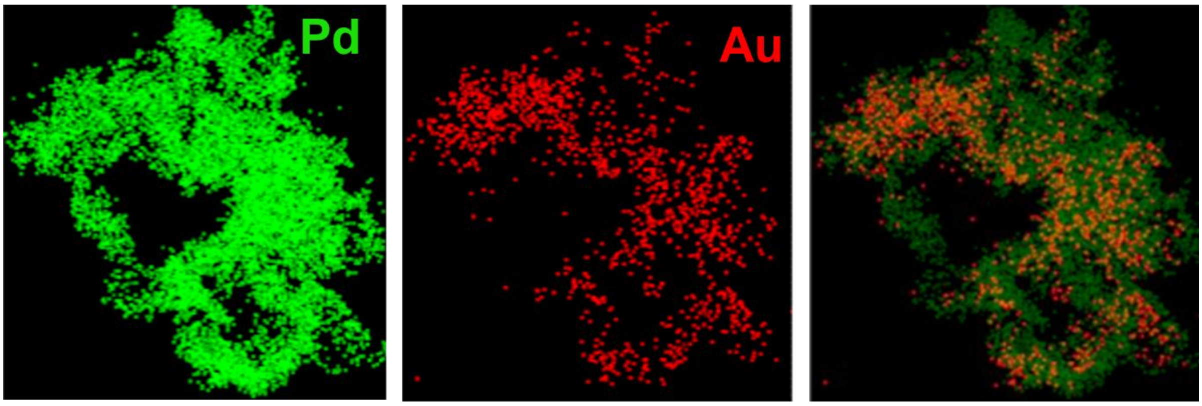

Figure S2: EDX of PdAu 10:1 NS’s.

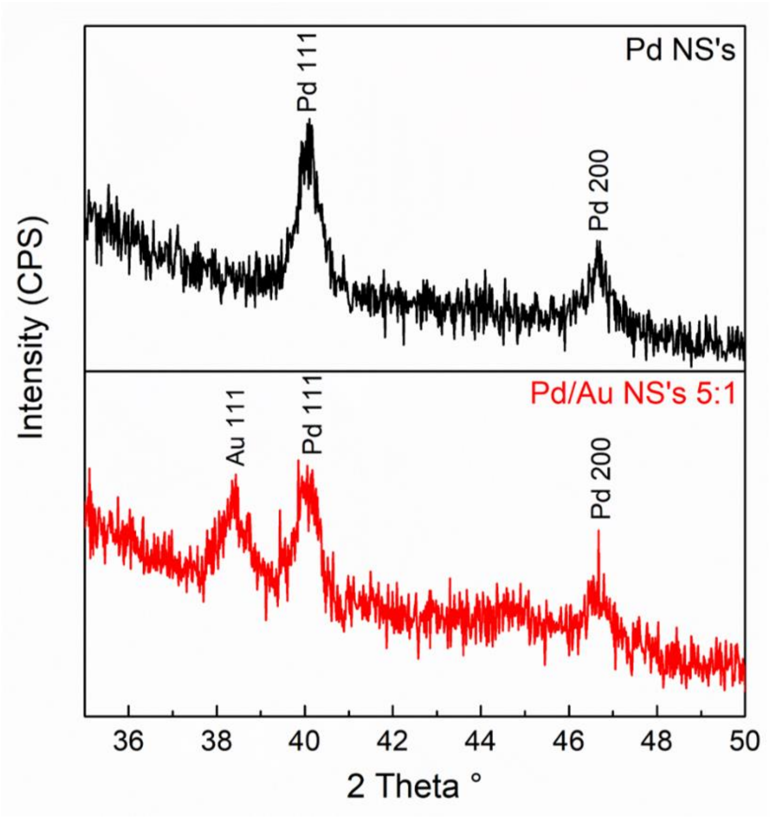

Figure S3: XRD of Pd and PdAu 5:1 NS’s

Moles of Pd	Moles of Au	Moles of surfactant	Moles of AA	Temp (c)	Time	Results
$1.6x10^{-5}$	-	$2.5x10^{-4}$	$3x10^{-4}$	0°C	6h	Nanoparticle formation
$1.6x10^{-5}$	-	$5x10^{-4}$	$3x10^{-4}$	0°C	6h	Nanoparticle formation
$1.6x10^{-5}$	-	$2.5x10^{-4}$	$3x10^{-4}$	0°C	3h	No reduction
$1.6x10^{-5}$	-	$5x10^{-4}$	$3x10^{-4}$	0°C	3h	No reduction
$8x10^{-6}$	-	$2.5x10^{-4}$	$3x10^{-4}$	0°C	6h	NS formation
$8x10^{-6}$	-	$5x10^{-4}$	$3x10^{-4}$	0°C	6h	NS and NP
$8x10^{-6}$	-	$2.5x10^{-4}$	$3x10^{-4}$	0°C	3h	NS and NP
$8x10^{-6}$	-	$5x10^{-4}$	$3x10^{-4}$	0°C	3h	NS and NP
$1.6x10^{-5}$	-	$2.5x10^{-4}$	$6x10^{-4}$	0°C	6h	No reduction
$8x10^{-6}$	-	$2.5x10^{-4}$	$6x10^{-4}$	0°C	6h	No reduction
$1.33x10^{-5}$	$2.66x10^{-6}$	$2.5x10^{-4}$	$3x10^{-4}$	0°C	6h	Nanoparticle formation
$1.33x10^{-5}$	$2.66x10^{-6}$	$5x10^{-4}$	$3x10^{-4}$	0°C	6h	Nanoparticle formation
$1.33x10^{-5}$	$2.66x10^{-6}$	$2.5x10^{-4}$	$3x10^{-4}$	0°C	3h	NS and NP
$1.33x10^{-5}$	$2.66x10^{-6}$	$5x10^{-4}$	$3x10^{-4}$	0°C	3h	NS and NP
$6.65x10^{-6}$	$1.35x10^{-6}$	$5x10^{-4}$	$3x10^{-4}$	0°C	6h	NS and NP
$6.65x10^{-6}$	$1.35x10^{-6}$	$2.5x10^{-4}$	$3x10^{-4}$	0°C	6h	NS formation
$6.65x10^{-6}$	$1.35x10^{-6}$	$2.5x10^{-4}$	$3x10^{-4}$	0°C	3h	NS and NP
$6.65x10^{-6}$	$1.35x10^{-6}$	$5x10^{-4}$	$3x10^{-4}$	0°C	3h	NS and NP
$1.33x10^{-5}$	$2.66x10^{-6}$	$2.5x10^{-4}$	$6x10^{-4}$	0°C	6h	No reduction
$6.65x10^{-6}$	$1.35x10^{-6}$	$2.5x10^{-4}$	$6x10^{-4}$	0°C	6h	No reduction
$7.28\ x10^{-6}$	$7.2\ x10^{-7}$	$2.5x10^{-4}$	$3x10^{-4}$	0°C	6h	NS formation
$1.456x10^{-5}$	$1.44x10^{-6}$	$5x10^{-4}$	$3x10^{-4}$	0°C	6h	NS and NP
$1.456x10^{-5}$	$1.44x10^{-6}$	$2.5x10^{-4}$	$3x10^{-4}$	0°C	3h	NS and NP
$1.456x10^{-5}$	$1.44x10^{-6}$	$5x10^{-4}$	$3x10^{-4}$	0°C	3h	NS and NP
$1.456x10^{-5}$	$1.44x10^{-6}$	$2.5x10^{-4}$	$6x10^{-4}$	0°C	6h	No reduction
$1.456x10^{-5}$	$1.44x10^{-6}$	$2.5x10^{-4}$	$6x10^{-4}$	0°C	6h	No reduction
$8x10^{-6}$	-	$2.5x10^{-4}$	$3x10^{-4}$	35°C	6h	No reduction
$6.65x10^{-6}$	$1.35x10^{-6}$	$5x10^{-4}$	$3x10^{-4}$	35°C	6h	No reduction
$1.456x10^{-5}$	$1.44x10^{-6}$	$2.5x10^{-4}$	$3x10^{-4}$	0°C	6h	No reduction

Table S1: Reaction parameters

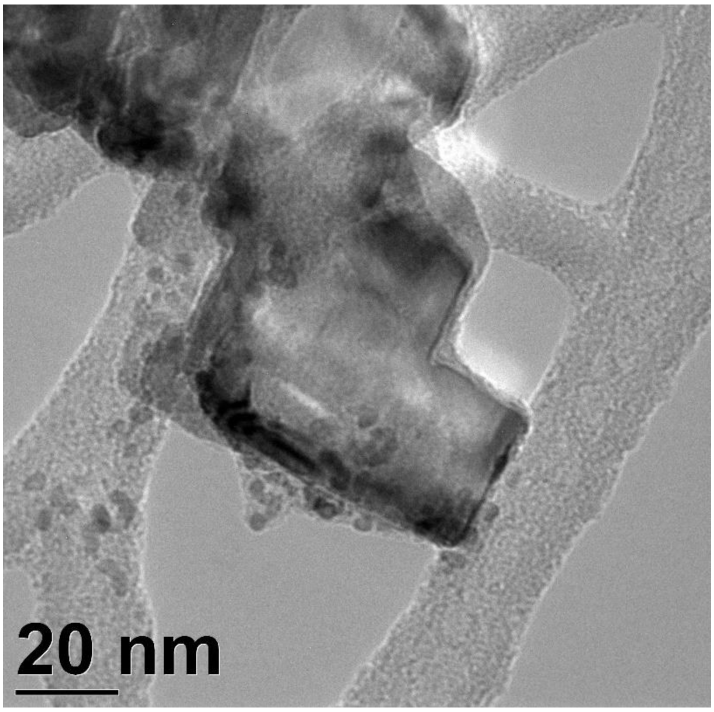

Figure S4: TEM image of PdAu 2:1 NS’s.

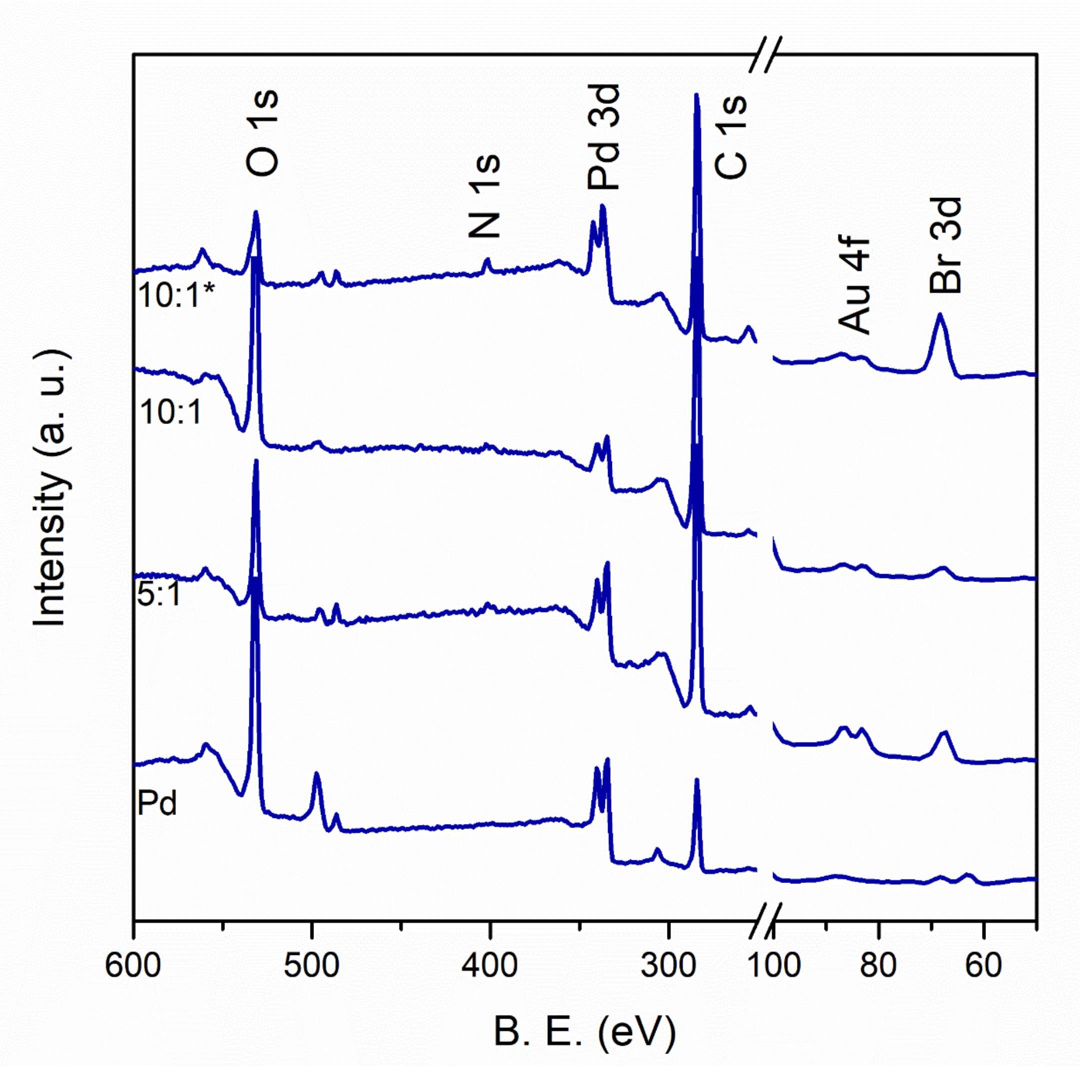

Figure S5: Survey scan of Pd NS, PdAu 5:1 NS and PdAu 10:1 NS with incomplete and complete reduction.

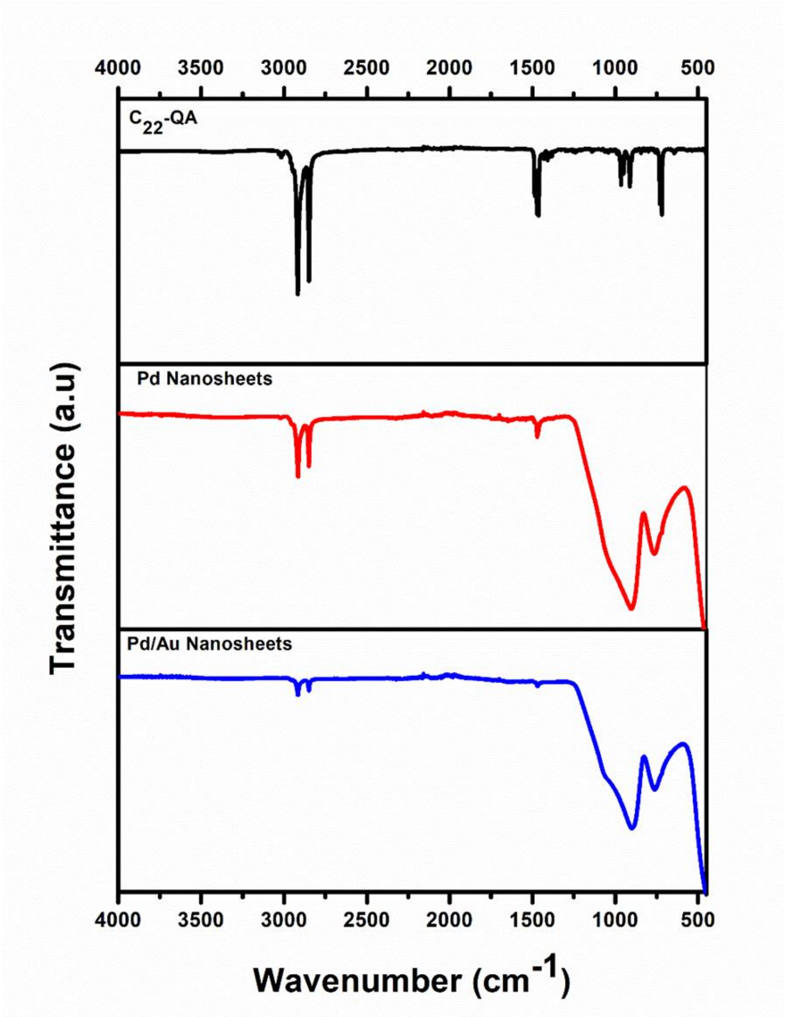

Figure S6: FTIR of the surfactant C_{22}-QA(Br^-), Pd and PdAu nanosheets.

Catalyst	Surface Ligand	Reaction conditions	% Yield	Ref
**Conventional Suzuki Cross Coupling**				
PdAu nanoflower	PVP	80°C, 15 min, 0.3 wt%	93 %	1
Pd Nanocube	CTAB	Room temperature, 30 min, 4 wt%,	95 %	2
AuPd Nanoparticles	N/A	30 °C, 6 h, 3 wt%	96 %	*3*
Pd NP-TiO_2		28 °C, 4 h, 5 wt%	93 %	*4*
Pd nanoparticles	PVA	60 °C, 30 min, 0.2 mol%	79 %	*5*
**Light-driven Suzuki Cross Coupling**				
PdAu NPs on ZrO_2		45°C, 24h , Visible light	80 % light<br>10 % Dark	6
Pd NP on 2H-WS_2	PVP	RT, 3h, 60 W LED, 2.85 µg catalyst	90%	*7*
Pd/ZnO		RT, 1.5 h, visible light	96 % Light<br>Trace Dark	*8*
AuPd nanowheels		50 °C, 90 min, Xe lamp, 0.2 mg catalyst	100 % Light<br>18 % dark	*9*
Pd hexagonal nanoplates	PVP	25°C, 3 h, Xe lamp<br>0.005 mmol catalyst loading	90 %	*10*
Pd/Au nanosheets	C_{22}-QA	0°C, 6h, Visible light, 0.2 mol% catalyst loading	99%	*This work*

Table S2**:** Summary of literature reports of catalysts used for Suzuki cross coupling under conventional (thermal) and light driven catalysis.

While it can be difficult to make direct comparison on catalytic performance, due to different reaction conditions (catalyst loading, time, temperature, light source), Table 1 is nevertheless useful.

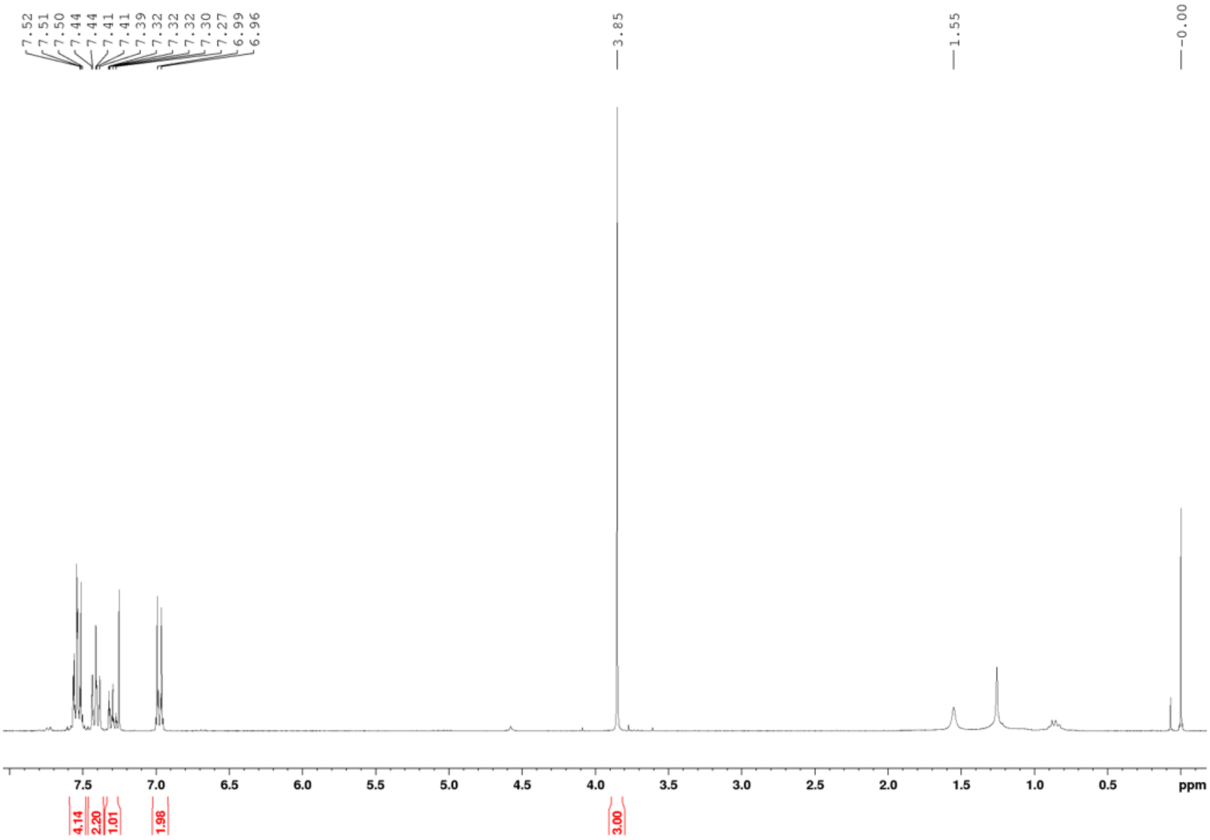

Figure S7: NMR of 4-methoxybiphenyl from the Suzuki cross couple reaction.

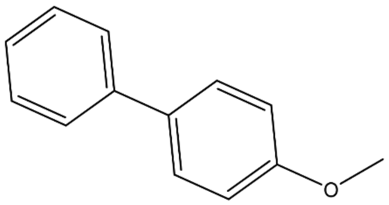

4-methoxybiphenyl: white solid, ^{1}H NMR (300 MHz $CDCl_3$)

δ= 7.56-7.55 (m, 4H), 7.41 (t, J=7.35Hz, 1H), 6.99 (d, J=8.8 Hz, 2H), 3.85 (s, 3H) ppm all in agreement with literature reports.

Catalyst	mol %	°C	Time	% Conversion	TON[a]	TOF(h^{-1})[b]
Pd Dark	0.1	25	2 h	0	0	0
Pd LED	0.1	25	2h	10	100	50
Pd Dark	0.2	25	2h	11	55	27.5
Pd LED	0.2	25	2h	10	50	25
PdAu Dark	0.1	25	2h	48	480	240
PdAu LED	0.1	25	2h	58	580	240
PdAu Dark	0.2	25	2h	36	180	90
PdAu LED	0.2	25	2h	93	465	232.5

Table S3: TOF for Pd and PdAu catalysts in the dark and under LED light at 0.1 and 0.2 mol% where [a] TON= moles of product x conversion/moles of Pd) and [b] TOF= TON/time (h)

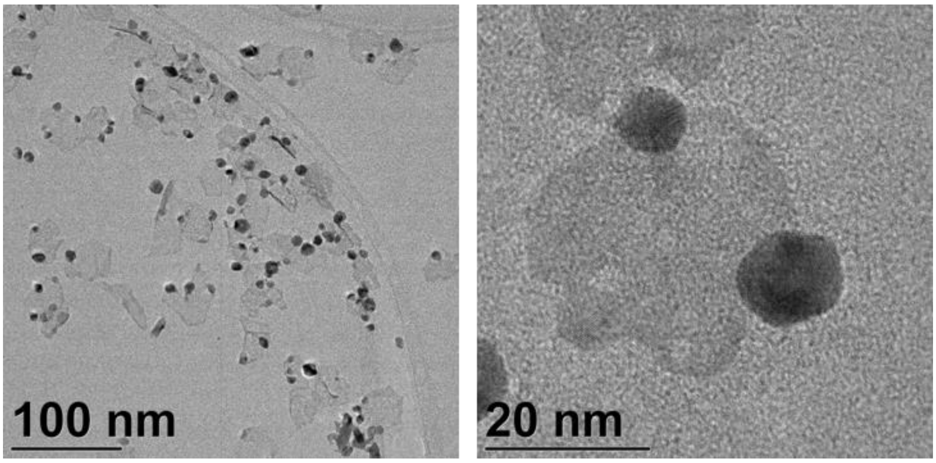

Figure S8: TEM images of CO-assisted Pd/Au nanosheets

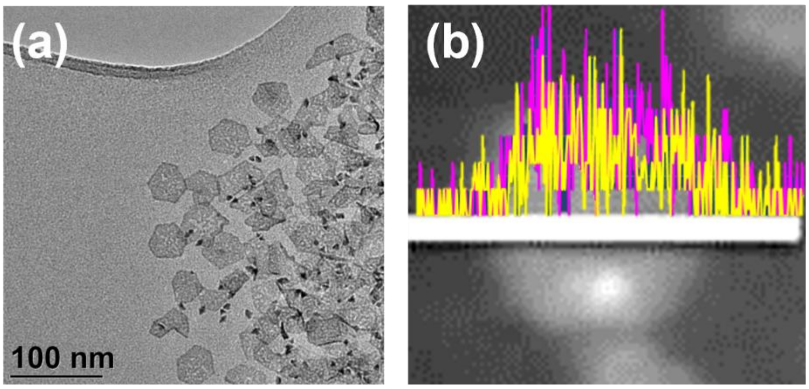

Figure S9: (a)TEM image and (b) EDX line scan of PdAg NS.

The CO-assisted methodology was successful for PdAg NSs. Supporting information, Figure S9 (a) shows a TEM image of PdAg NSs with a well-defined morphology and a mean length of 24 nm. shows a single PdAg NS and alloy formation was confirmed by EDX analysis, as displayed in Figure 9 (b), showing a homogenous distribution of Pd and Ag across the NS.

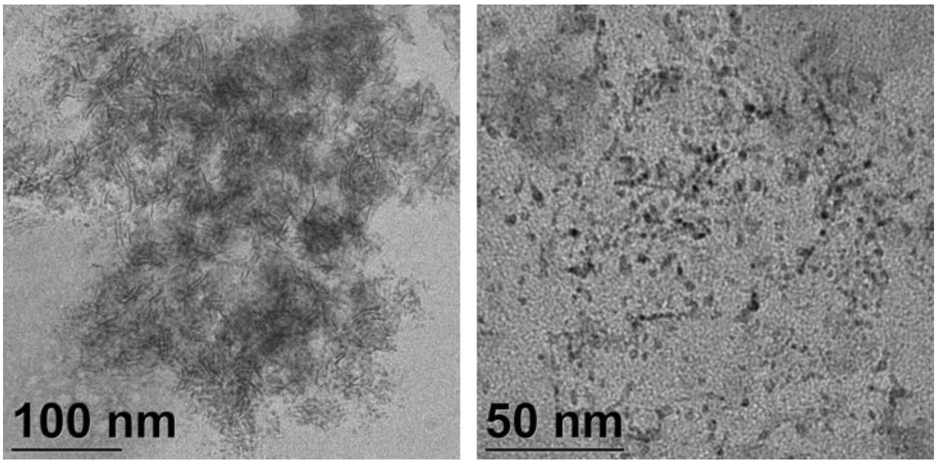

Figure S10: TEM images of nanosheets which were destroyed by chemically cleaned using $NaBH_4$

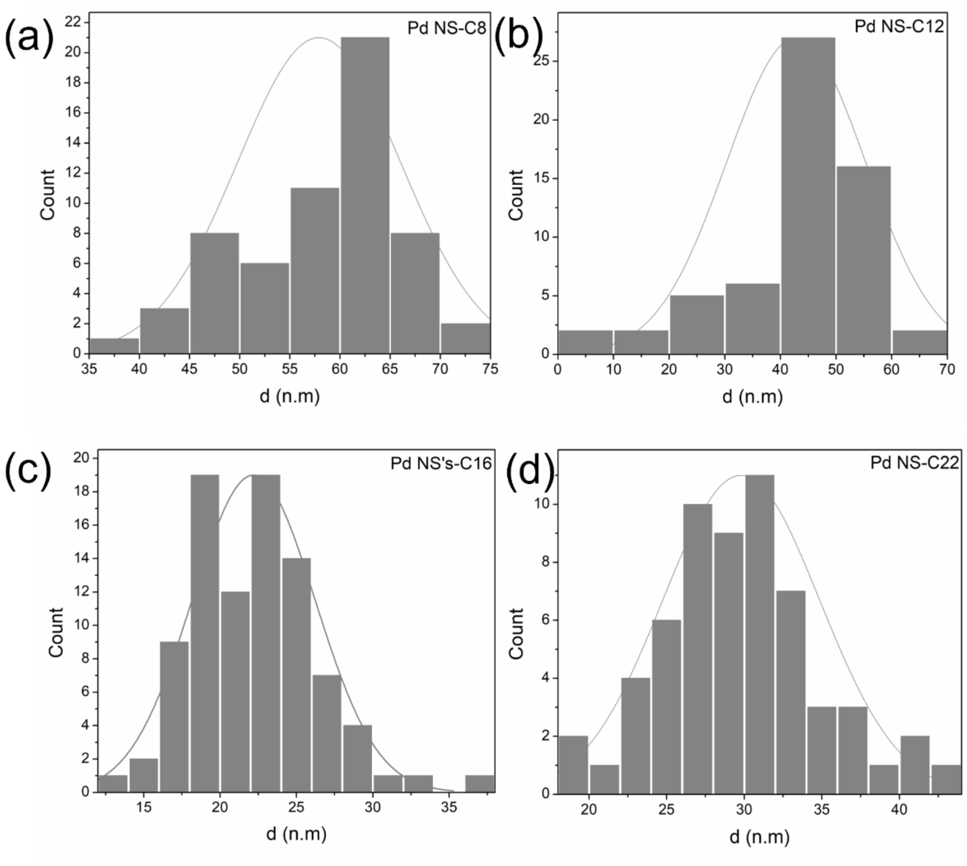

Figure S11: Size distribution profile of Pd NS with chain lengths (a) C8, (b) C12, (c) C16 and (d) C22

References

1. Xu, J.; Wilson, A. R.; Rathmell, A. R.; Howe, J.; Chi, M.; Wiley, B. J., Synthesis and Catalytic Properties of Au–Pd Nanoflowers. *ACS Nano* **2011,** *5* (8), 6119-6127.
2. Swain, S.; M. B, B.; Kandathil, V.; Bhol, P.; Samal, A. K.; Patil, S. A., Controlled Synthesis of Palladium Nanocubes as an Efficient Nanocatalyst for Suzuki–Miyaura Cross-Coupling and Reduction of p-Nitrophenol. *Langmuir* **2020,** *36* (19), 5208-5218.
3. Xiao, Q.; Sarina, S.; Jaatinen, E.; Jia, J.; Arnold, D. P.; Liu, H.; Zhu, H., Efficient photocatalytic Suzuki cross-coupling reactions on Au–Pd alloy nanoparticles under visible light irradiation. *Green Chemistry* **2014,** *16* (9), 4272-4285.
4. Koohgard, M.; Hosseini-Sarvari, M., Enhancement of Suzuki–Miyaura coupling reaction by photocatalytic palladium nanoparticles anchored to TiO2 under visible light irradiation. *Catalysis Communications* **2018,** *111*, 10-15.
5. Chatterjee, S.; Bhattacharya, S. K., Size-Dependent Catalytic Activity and Fate of Palladium Nanoparticles in Suzuki-Miyaura Coupling Reactions. *ACS Omega* **2018,** *3* (10), 12905-12913.
6. Xiao, Q.; Sarina, S.; Bo, A.; Jia, J.; Liu, H.; Arnold, D. P.; Huang, Y.; Wu, H.; Zhu, H., Visible Light-Driven Cross-Coupling Reactions at Lower Temperatures Using a Photocatalyst of Palladium and Gold Alloy Nanoparticles. *ACS Catalysis* **2014,** *4* (6), 1725-1734.
7. Raza, F.; Yim, D.; Park, J. H.; Kim, H.-I.; Jeon, S.-J.; Kim, J.-H., Structuring Pd Nanoparticles on 2H-WS2 Nanosheets Induces Excellent Photocatalytic Activity for Cross-Coupling Reactions under Visible Light. *Journal of the American Chemical Society* **2017,** *139* (41), 14767-14774.
8. Hosseini-Sarvari, M.; Bazyar, Z., Visible Light Driven Photocatalytic Cross-Coupling Reactions on Nano Pd/ZnO Photocatalyst at Room-Temperature. *ChemistrySelect* **2018,** *3* (6), 1898-1907.
9. Huang, X.; Li, Y.; Chen, Y.; Zhou, H.; Duan, X.; Huang, Y., Plasmonic and catalytic AuPd nanowheels for the efficient conversion of light into chemical energy. *Angewandte Chemie (International ed. in English)* **2013,** *52* (23), 6063-7.
10. Trinh, T. T.; Sato, R.; Sakamoto, M.; Fujiyoshi, Y.; Haruta, M.; Kurata, H.; Teranishi, T., Visible to near-infrared plasmon-enhanced catalytic activity of Pd hexagonal nanoplates for the Suzuki coupling reaction. *Nanoscale* **2015,** *7* (29), 12435-12444.
